# Supplementary material for: Effectiveness of Bioactive Compound as Antibacterial and Anti-Quorum Sensing Agent from Myrmecodia pendans: An In Silico Study
Source: Molecules. 2021 Apr 23;26(9):2465. doi: 10.3390/molecules26092465 (PMC8122932; doi:10.3390/molecules26092465)
Supplement: Supplementary file 1 [file molecules-26-02465-s001.zip › molecules-1099564-supplementary.pdf]

Supplement material Table S1. Sum and hydrogen bond of complex in PBP, MurB, SrtA and DNA gyrase

No	Compound	PBP	MurB	SrtA	DNA gyrase
1	Compound **1**	14 ; Arg233, Glu368(**2**), Glu193, Lys158, Ser232(**4**), Tyr235, Asn358, Asn362(**3**),	4; Ser58(**2**), Tyr133, Ile182	3 ; Asp137, Asn127(2)	5 ; Gln85, Glu52, Asp75, Gly79, Thr167
2	Compound **2**	3 ; Ser232, Lys197A, Asn362A	9; Ser66, Arg209(2), Lys212, Gln242, Thr232, Ser222(2), Gly221	4 ; Val69, Ala70, Ala72, Ser105	2; Ala86, Thr167
3	Compound **3**	7; Asp285(**2**), Lys197(**2**), Thr265, Arg143, Val267,	5; Tyr139, Lys212, Gln213, Asn67, Ser222	6 ; Cys187(2), Ala58, Met56, Glu112, Ser59	3 ; Glu52, Asp75, Thr167
4	Compound **4**	3; Met360A, Trp202, Asn362A	3; Arg226, Gln213, Arg209	3; Gly53, Met56, Asp57	2; Asp75, Thr167
5	Compound **5**	3; Glu193, Asn362, Ser232	2; Ser127, Arg294	6 ; Asn127, Ile138, Ala139, Lys195, Lys137, Ser194	2 ; Asn48, Pro81
6	Compound **6**	3; Lys197, Asn362, Pro210	2; Glu292, Ser222	4 ; Ser59, Lys187, Ser54, Ile61	2 ; Pro232, Tyr258
7	Compound **7**	-	2; Trp59, Tyr133	1 ; Gly53	2 ; Glu52, Asn48
8	Compound **8**	1; Thr265	4; Asn23, Asn64, Gly126, Lys28	2; Glu112, Ser98	2; Tyr29, Glu44
10	Carbapenems	4; Pro210, Trp202, Asn362, Lys197	NT	NT	NT
11	Glycopeptides	NT	9; Tyr139, Arg209(**2**), Lys12(**2**), Ser222(**2**), Gly137, Gly233	NT	NT

No	Compound	PBP	MurB	SrtA	DNA gyrase
12	Quercetin	NT	2; Trp59, Arg294	NT	NT
13	Amoxicillin	NT	NT	6 ; Met56, Asp57, His55, Lys60, Ile61, Ser49	NT
14	Cefixime	NT	NT	6 ; Lys96, Val110, Ser98(2), Glu112, Ser97	NT
15	Curcumin	NT	NT	2 ; Asp137, Ser97	NT
16	Gatifloxacin	NT	NT	NT	2 ; Asp75, Asn48

Supplement material Table S2. Hydrophobic contact of complex in PBP, MurB, SrtA and DNA gyrase

No	Compound	PBP	MurB	SrtA	DNA gyrase
1	Compound **1**	2; Phe160, Lys359	3; Tyr133, Trp59(**2**)	4; Tyr197, Ala139, Val190, Lys195	4; Pro81, Ala92, Val96,Ile80
2	Compound **2**	3; Lys197, Val267, Arg143,	1; Phe231	-	6; Ile80, Pro81, Val96, Ala92, Asn48, Ser49
3	Compound **3**	4; Trp202(**2**), Val267, Arg143	5; Tyr139(**2**), Ile124, Ala138, Ala136	3; Trp171, Ala58, Lys111	4; Ile80, Val96, Pro81, Ala92
4	Compound **4**	2; Trp202A, Ile364	8; Ile124(**3**), Ala136(**2**), Pro125, Leu103, Ala131,	2; Lys60, Ala58	5; Asn48, Ser49, Ile80, Pro81, Val96
5	Compound **5**	3; Asn358, Lys359, Val213	4; Val61(**2**), Tyr133(**2**)	3; Tyr197, Ala139, Val190	-
6	Compound **6**	5; Lys197(**2**), Pro210, Trp202(**2**)	5; Ala138, Pro125, Ala138, Tyr139, His255	-	2; Ile234, Leu316

No	Compound	PBP	MurB	SrtA	DNA gyrase
7	Compound **7**	8; Trp202(**5**), Pro210(**3**)	12; Leu60(**3**), Trp59(**3**), Ile296, Tyr133(**3**), His180(**2**)	-	1; Ile80
8	Compound **8**	4; Val267, Arg143, Arg233, Lys197	4; Ile104, Lys102, Leu215, Tyr108	1; Ala58	3; Ile80, Pro81, Val96
9	Penicillin	2; Ile364, Pro210	NT	NT	NT
10	Carbapenems	4; Trp202(**3**), Pro210	NT	NT	NT
11	Glycopeptides	NT	1; Lys234	NT	NT
12	Quercetin	NT	5; Tyr133(**2**), Trp59, Val61(**2**)	NT	NT
13	Amoxicillin	NT	NT	-	NT
14	Cefixime	NT	NT	-	NT
15	Curcumin	NT	NT	3; Tyr197, Lys195, Ala139	NT
16	Gatifloxacin	NT	NT	NT	2; Pro81, Val96
17	Sitofloxacin	NT	NT	NT	2; Ile80, Pro81

Supplement material Table S3. Sum and hydrogen bond of complex in RNA polymerase and ribosomal subunit

No	Compound	RNA polymerase subunit		Ribosomal	
		alfa	beta	30S	50S
1	Compound **1**	4 ; Pro7, Thr216(2), Ala212	3 ; Arg1050, Gly1022, Asn1046	3 ; Gly62, Arg58, Arg108	1 ; Leu28
2	Compound **2**	2; Val171, Thr198	2 ; Ala598, His605	3 ; Thr5,Arg109, Leu19	1 ; Lys24
3	Compound **3**	6 ; Lys86, Ser114, Ser201, Ala49, Tyr88, Gly48	4 ; Cys892, Arg1077, Lys895, Arg822	5 ; Arg101, Arg108, Gly161, Ala105, Ser20	3 ; Lys24, Arg20, Gly29
4	Compound **4**	2; Glu91, Leu87	1 ; Asp840	5 ; Ser8(2), Thr107, Gly6, Pro7	2 ; Val23, Gly29
5	Compound **5**	1 ; Ala49	-	2 ; His59, Ser20	1 ; Val23
6	Compound **6**	1 ; Glu91	1; Met241	2 ; Gln138, Asp128	1 ; Gly29
7	Compound **7**	1 ; Thr216	2 ; Pro232, Tyr258	1 ; Ala105	1 ; Gly29
8	Compound **8**	1; Ile9	3; Tyr106, Gly13,HIs103	4 ; Ser20(2), Arg101, Arg108	-
9	Myxopyronin	2 ; Thr216	2 ; Arg642	NT	NT
10	Rifamycin	2 ; Glu91, Glu206	1 ; Ser85	V	NT
11	Tetrasiklin	NT	NT	5 ; Arg3, Arg14, Arg109, His59, Gly62	NT
12	Chloramphenicol	NT	NT		1 ; Gly29

Supplement material Table S4. Hydrophobic contact of complex in RNA polymerase and ribosomal subunit

No	Compound	RNA polymerase subunit		Ribosomal	
		alfa	beta	30S	50S
1	Compound **1**	3; Ala212, Val23, Leu208	5; Leu418, Ser797, Gly798, Tyr799, Arg802	3; Arg108, Arg109, Val11	4 ; Arg20, Ala21, Val23, Lys24
2	Compound **2**	3; Leu43, Leu172, Ile167	1; Leu1042	5;2Val11, His59, Arg108, Arg109	7; 3Lys24, Val12, Arg20, Val35,Lys37
3	Compound **3**	5; Tyr88, Ser201, Ile202, Ala49, Lys86	3; Phe890, Val1041, Ala1098	2; Val160, Leu19	2; Val23, Lys24
4	Compound **4**	2; Ala49, Pro142	2; Ile606, Ala873	2; Val160, Leu186	2; Val23, Lys24
5	Compound **5**	2; Ala84, Ile167	1; Tyr353	2; Val11, His59	2 ; Lys24, Arg20
6	Compound **6**	-	-	3 ; Lys125, Tyr132, Ile129	4 ; Arg20, Lys24, Val12, Val23
7	Compound **7**	1; Lys6	2; Ile234, Leu316	3 ; Tyr100, Val160, Ala158	-
8	Compound **8**	4; Lys6, Arg8, Pro7, Leu34	4; Leu316, Ile234, Leu331, Arg326	7; Ala164, Pro163, Pro181, Phe165, 2Leu186, Ala158	4; Arg20, Ala21, 2Val12,
9	Myxopyronin	4; Pro7, Leu34, Ile9, Tyr30	5; Tyr353, Leu474, Pro409, Arg70, Ala476	NT	NT
10	Rifamycin	2; Pro47, Pro142	-	NT	NT
11	Tetrasiklin	NT	NT	1 ; Lys24	NT
12	Chloramphenicol	NT	NT	NT	1 ; Lys24

Supplement material Table S5. Sum and hydrogen bond of complex in protein QS

No	Compound	ClyM	FsrB	GBAP	PgrX
1	Compound **1**	5; Asn217A, Ser261A, Met221A, Ile143A, Phe214A	4; Glu129A, Asn137A, Ser143A, Tyr121A	3; Gln43A, Glu45A, Thr44A	3; Glu279, Thr199, Tyr165
2	Compound **2**	4; Lys274A, Glu313A, Asp364A, Thr512A	2; Asn137A, Ser143A	2; Met41A, Glu45A	2; Asn63, Lys184
3	Compound **3**	4; Trp84A, Ala531A, Tyr213A, His553A	1; Glu129A	3; Gly38A, Gly42A, Ile36A	3 ; Ser111, Asn73, Gln108
4	Compound **4**	4; Thr259A, Glu520A, His150A, Gly251A	2; Gln124A, Ala127A	3; Gly42A, Gln43A, Thr44A	2; Ser118, Asn161
5	Compound **5**	5; Arg210A, Phe214A, Thr218A, Gly251A, Ser249A	4; Trp120A, Glu129A, Tyr121A, Ser143A	2; Trp40A, Thr44A	5 ; Ser118, Gly115, Ile112, Ser111, Glu154
6	Compound **6**	2; Gln250A, Ile315A	1; Lys96A	1; Asn35A	3 ; Gly150, Tyr186, Phe182
7	Compound **7**	1; Lys276A	1; Lys77A	2; Gln31A, Asn32A	1 ; Met67
8	Compound **8**	3; Lys388A, Phe667A, Ala665A	3; Tyr88A, Lys96A, Leu149A	3; Ser33A, Asn35A, Arg15A	2; Asn73, Thr69
9	Amoxicillin	NT	NT	NT	4 ; Ser83, Glu154, Ser111, Asn158
10	Cefixime	NT	NT	NT	4 ; Ser111, Asn161(2), Ser118
11	(+)-AMP	9; Tyr95A, Asn96A, Glu99A, Gln116A, Arg119A, Arg112A, Glu104A, Asp106A, Ser98A	NT	NT	NT
12	Ambuic acid	NT	2; Tyr88A, Leu152A	2; Tyr88A, Leu152A	NT

Supplement material Table S6. Hydrophobic contact of complex in protein QS

No	Compound	ClyM	FsrB	GBAP	PgrX
1	Compound **1**	1; Thr259A	3; Leu136A, Tyr121A, Lys140A	1; Trp40A	2; Thr199, Ile200
2	Compound **2**	4; Val260A, Val272A, His349A, Ile63A	5; Phe118A, Pro123A, Val115A, Ile111A, Leu114A	3; Trp40A, Met41A, Gly42A	2; Phe182, Phe59
3	Compound **3**	4; Ile560A, Tyr213A, Ala513A, Val209A	3; Ala122A, Tyr121A, Lys140A	4; Trp40A, Gly38A, Gln39A	3; Ile82, Lys79(2) , Lys70,
4	Compound **4**	2; Ile143A, Phe214A	5; Trp78A, Arg81A, Lys77A, Leu139A, Trp120A	3; Trp40A, Gln43A, Thr44A	3; Lys79, Ile82, Ile112
5	Compound **5**	2; Thr259A, Ile143A	3; Leu136A, Lys140A, Leu144A	1; Trp40A	1; Ile82
6	Compound **6**	-	-	-	-
7	Compound **7**	4; Val272A, Ile354A, Ile363A, Tyr314A	1; Leu139A	-	1; Lys79
8	Compound **8**	8; Arg664A, Lys388A, Ile610A, Ile677A, Leu961A, Pro374A, Tyr387A, Tyr588A	8; Val89A, Pro92A, Ile85A, Leu150A, Leu82A, Trp78A, Tyr88A, Phe93A	2; Trp40A, Phe37A	2; Phe149, Leu107
9	Amoxicillin	NT	NT	NT	1; Phe86
10	Cefixime	NT	NT	NT	1; Ile82
11	(+)-AMP	-	NT	NT	NT
12	Ambuic acid	NT	5; Val116A, Leu150A, Ile85A, Tyr88A, Trp120	5; Val116A, Leu150A, Ile85A, Tyr88A, Trp120	NT
